# Prediction of Optimal Positive Airway Pressure in Chinese Patients With Obstructive Sleep Apnea

**DOI:** 10.1111/crj.70047

**Published:** 2024-12-23

**Authors:** Feng Pang, Wenmin Deng, Jingyan Huang, Yu Guo, Minmin Lin, Xiangmin Zhang, Jie Liu

**Affiliations:** ^1^ Department of Sleep Medicine The Sixth Affiliated Hospital, Sun Yat‐sen University Guangzhou Guangdong China; ^2^ Department of Otolaryngology–Head and Neck Surgery The Sixth Affiliated Hospital, Sun Yat‐sen University Guangzhou Guangdong China; ^3^ Biomedical Innovation Center The Sixth Affiliated Hospital, Sun Yat‐sen University Guangzhou China; ^4^ Department of Otolaryngology–Head and Neck Surgery Foresea Life Insurance Guangzhou General Hospital Guangzhou Guangdong China

**Keywords:** China, obstructive sleep apnea (OSA), optimal pressure, positive airway pressure (PAP), prediction

## Abstract

**Purpose:**

Positive airway pressure (PAP) is the primary treatment for obstructive sleep apnea (OSA). This study aims to predict the optimal PAP pressure in Chinese OSA patients by their polysomnography (PSG) variables and demographic characteristics.

**Methods:**

Patients with an apnea–hypopnea index (AHI) ≥ 15 times/h who received PAP therapy (residual AHI < 5 times/h) and underwent PSG were included in this study. Sex, age, body mass index (BMI), Epworth Sleepiness Scale (ESS), AHI, supine AHI, lowest oxygen saturation (LSaO_2_), percentage of total sleep time spent with SaO_2_ < 90% (CT90), and PAP pressure were recorded. PAP pressure and other variables were analyzed using univariate correlation and multivariate linear stepwise regression analysis.

**Results:**

A total of 167 patients were enrolled, with 122 in the study group and 45 in the validation group. Univariate correlation analysis revealed a significant correlation between PAP pressure and age, BMI, ESS, AHI, supine AHI, LSaO_2_, and CT90. The multivariate linear regression analysis showed that PAP pressure was correlated with gender (*b* = 1.142, *p* = 0.032), age (*b* = −0.039, *p* = 0.005), AHI (*b* = 0.047, *p* = 0.000), and CT90 (*b* = 0.037, p = 0.000). The final PAP pressure prediction equation was PAPpre (cmH_2_O) = 8.548 + 1.142 × sex −0.039 × age + 0.047 × AHI + 0.037 × CT90 (*R* ^2^ = 0.553) (male is defined as 0 and female as 1). This model accounts for 55.3% of the optimal pressure variance, and the area under the ROC curve of PAP prediction pressure is 0.7419.

**Conclusion:**

PSG variables can be used to predict PAP pressure in Chinese OSA patients, but for some individuals, the prediction model is not very good. PAP is correlated with age, BMI, ESS, AHI, supine AHI, LSaO_2_, and percentage of total sleep time spent with SaO_2_ < 90% (CT90), which can be used to predict the optimal PAP pressure.

## 1 Introduction

Obstructive sleep apnea (OSA) is a sleep disorder characterized by repeated upper airway collapse leading to apnea or hypopnea [1]. It often causes snoring, daytime sleepiness, dry mouth in the morning, and decreased concentration [2]. OSA is associated with an increased incidence of hypertension, diabetes, and cardiovascular disease [3, 4, 5]. In the United States, the prevalence rate is 14% for middle‐aged men and 5% for middle‐aged women [6].

Currently, positive airway pressure (PAP) is the standard treatment for moderate to severe OSA [7]. However, approximately 50% of patients have poor adherence to continuous positive airway pressure (CPAP) therapy [8]. Adherence is thought to be influenced by the first few days of therapy and the initial PAP pressure [9, 10]. High pressure can cause dry mouth and poor sleep, whereas low pressure is less effective. There are three methods for selecting PAP pressure: (1) PAP titration, which is time‐consuming and costly and must be done in a laboratory; (2) automatic PAP pressure selection (APAP), which can be done at home but has poorer adherence than PAP titration [7]; and (3) using a PAP pressure prediction equation, which is a simple and time‐saving method for predicting the optimal PAP pressure [11]. By using the formula to predict the optimal PAP pressure, patients can quickly obtain the appropriate pressure and improve adherence to PAP therapy.

The PAP pressure prediction formula is widely used in many countries and regions. However, because of differences in race and physiological characteristics, the optimal PAP pressure prediction equation varies by country. Moreover, the current forecasting companies face challenges such as low accuracy, reliance on single‐center data, and some formula variables that are difficult to obtain [12]. Therefore, the aim of this study was to develop a simple and accurate PAP pressure prediction formula tailored to the Chinese population.

## 2 Material and Methods

**FIGURE 1:**
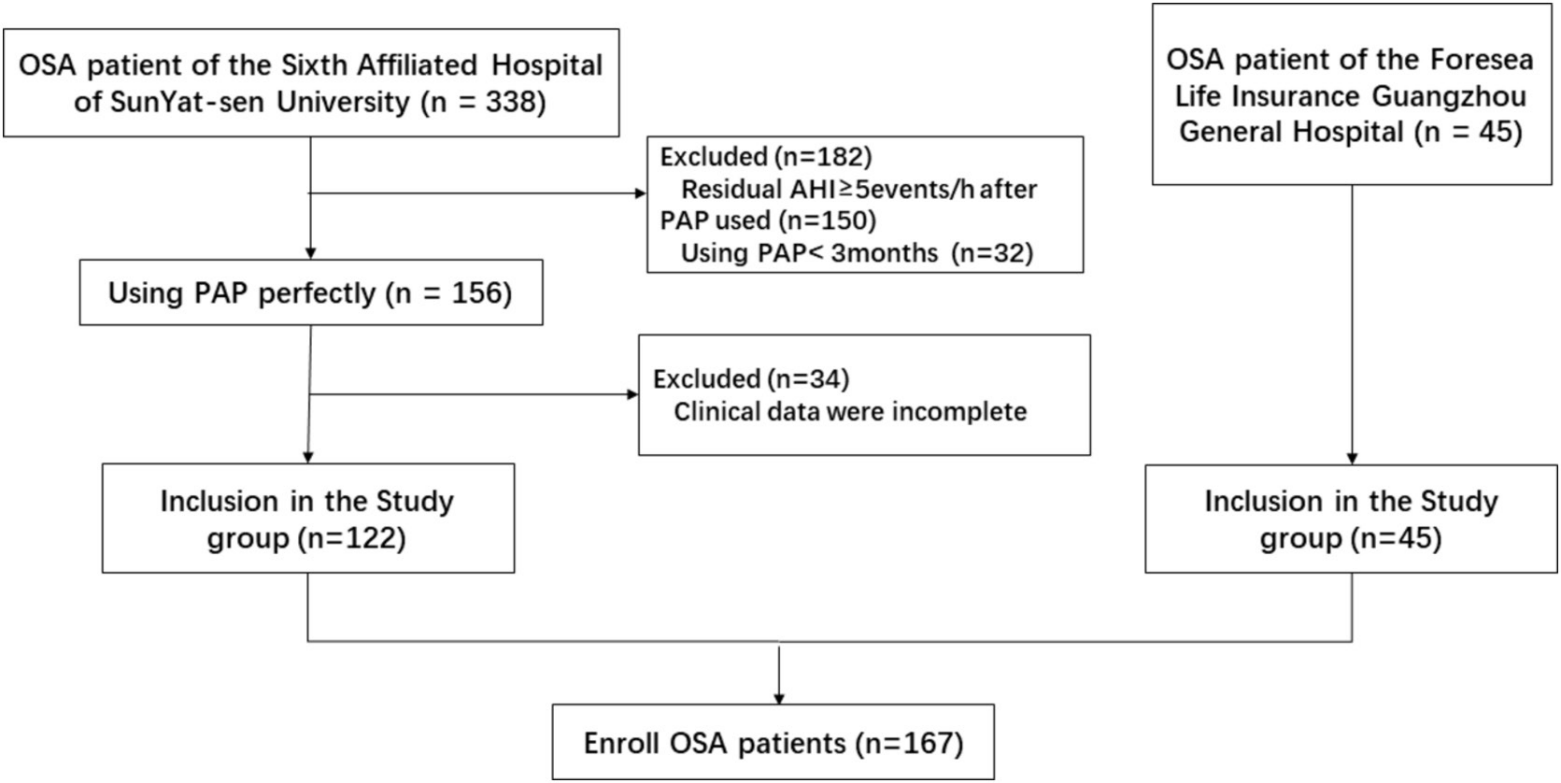
Study flow chart.

This is a retrospective study that was approved by the Human Research Ethics Committee of the Sixth Affiliated Hospital of Sun Yat‐sen University (2021ZSLYEC‐385) and conducted in accordance with the Declaration of Helsinki. As the study was retrospective in nature, individual consent from study participants was not obtained. The study included adult OSA patients who underwent polysomnography (PSG) and PAP therapy between April 2015 and October 2021 at the Sleep Medicine Center of the Sixth Affiliated Hospital of Sun Yat‐sen University and the Foresea Life Insurance Guangzhou General Hospital. Figure 1 shows the flow chart.

### 2.1 Inclusion Criteria

The inclusion criteria are as follows: (1) age ≥ 18 years; (2) apnea–hypopnea index (AHI) ≥ 15 times/h, with the lowest oxygen saturation (LSaO_2_) ≤ 90% and previous PAP treatment; (3) residual AHI < 5 times/h after APAP treatment; (4) no history of OSA‐related surgery; and (5) using PAP more than 3 months.

### 2.2 Exclusion Criteria

Patients who had a long‐term use of known sleep‐disrupting drugs or current alcohol or drug abuse, congestive heart failure, obstructive pulmonary disease, a history of mental illness, or central sleep apnea syndrome were excluded.

### 2.3 Grouping

The study group consisted of OSA patients from the Sixth Affiliated Hospital of Sun Yat‐sen University, whereas other patients from Foresea Life Insurance Guangzhou General Hospital were enrolled in the validation group to confirm the accuracy of the prediction formula.

### 2.4 Observation Index

The following variables were observed: demographic information such as age, sex, height, weight, body mass index (BMI), self‐reported Epworth Sleepiness Scale (ESS), and history of hypertension, diabetes, and cardiopathy. PSG variables included AHI, supine AHI, LSaO_2_, and the percentage of total sleep time spent with SaO_2_ < 90% (CT90). Additionally, 90% APAP pressure was recorded.

### 2.5 PSG

The patients underwent a standard full‐night sleep laboratory examination at the Sleep Medicine Department of the Sixth Affiliated Hospital of Sun Yat‐sen University. The examination included electroencephalogram, electrocardiogram, blood oxygen, nasal airflow, chest and abdominal movement, body position, and snoring. The PSG results were manually analyzed in accordance with the American Academy of Sleep Medicine (AASM) standards [13]. Apnea was defined as a decrease in airflow of ≥ 90% from baseline for at least 10 s, whereas hypopnea was defined as a reduction of nasal pressure of ≥ 30% from baseline for at least 10 s, with ≥ 3% oxygen desaturation or accompanied by an EEG arousal for 3–15 s. Supine AHI referred to the AHI in the supine position, whereas CT90 represented the percentage of total sleep time spent with SaO_2_ < 90%.

### 2.6 PAP Titration

All subjects in this study underwent nasal PAP titration to determine the optimal PAP pressure for treatment [14]. Specifically, all patients underwent APAP pressure titration in the laboratory for 3 days, and the optimal pressure was defined as the 90% APAP pressure when the residual AHI was less than five times per hour after APAP treatment.

### 2.7 Statistical Analysis

All statistical analyses were conducted using SPSS 24.0 for Windows and GraphPad Prism 8.0 for Windows. Pearson's correlation coefficient was used to examine the relationship between optimal pressure and continuous variables. A one‐way ANOVA test was used for categorical variables. Multiple linear regression with a backward stepwise method was performed to identify the independent predictive variables and establish the optimal PAP prediction equation. Paired *T*‐test was used to compare the predicted and measured pressures. The prediction formula was verified using ROC curve analysis. A *p*‐value of less than 0.05 was considered statistically significant.

## 3 Results

A total of 167 patients were enrolled in this study, with 122 in the study group and 45 in the validation group. The mean age of the participants was 44.34 ± 12.47 years, and their mean AHI, LSaO_2_, and PAP pressure were 46.86 ± 26.79 times/h, 69.46 ± 12.87%, and 10.63 ± 2.73 cmH_2_O, respectively. Table 1 provides a detailed description of the demographic and PSG examination results.

**TABLE 1:** Anthropometric variables and PSG variables.

Varible	Study group (Mean ± SD) (*n* = 122)	Validation group (*n* = 45)	Total (*n* = 167)
Males (*n*, %)	108 (88.52)	42 (93.3)	150 (89.8)
Females (*n*, %)	14 (11.48)	3 (6.7)	17 (10.2)
Age, years	44.34 ± 12.51	44.33 ± 12.52	44.34 ± 12.47
High, m	1.68 ± 0.71	1.69 ± 0.07	1.68 ± 0.07
Weigh, kg	77.165 ± 12.07	81.69 ± 15.88	78.38 ± 13.31
BMI, kg/m^2^	27.309 ± 3.90	28.51 ± 4.99	27.63 ± 4.24
ESS	9.16 ± 5.39	10.02 ± 5.02	9.39 ± 5.29
Hypertension (*n*, %)	38 (31.15)	10 (22.2)	48 (28.7)
Diabetes (*n*, %)	10 (8.2)	4 (8.9)	14 (8.4)
Cardiopathy (*n*, %)	13 (10.66)	4 (8.9)	17 (10.2)
AHI, times/h	44.183 ± 25.82	54.12 ± 28.28	46.86 ± 26.79
Supine AHI, times/h	49.546 ± 26.30	59.95 ± 27.65	52.35 ± 26.99
LSaO_2_, %	69.23 ± 13.10	70.07 ± 12.34	69.46 ± 12.87
CT90, %	15.830 ± 19.92	21.47 ± 22.30	17.35 ± 20.67
PAP pressure, cmH_2_O	10.741 ± 2.63	10.33 ± 2.97	10.63 ± 2.73

Abbreviations: AHI, apnea–hypopnea index; BMI, body mass index; CT90, the percentage of total sleep time spent with SaO_2_ < 90%; ESS, Epworth Sleepiness Scale; LSaO_2_, the lowest oxygen saturation; PAP, positive airway pressure.

**FIGURE 2:**
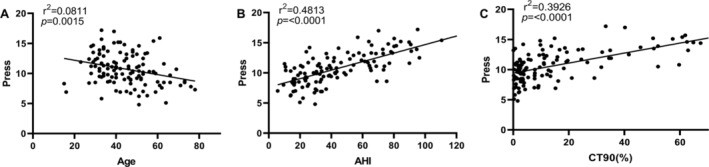
The correlations between PAP pressure and other variables through scatter plots, including (A) age, (B) AHI (apnea–hypopnea index), and (C) CT90 (the percentage of total sleep time spent with SaO_2_ < 90%).

As shown in Table 2 and Figure 2, PAP pressure in the study group was positively correlated with body weight, BMI, ESS, AHI, supine AHI, and CT90, whereas age and LSaO_2_ were negatively correlated with PAP pressure.

**TABLE 2:** Correlations between the optimal pressure and baseline characteristics, and polysomnographic parameters.

Variable	Correlation	*p*
Gender	0.407^a^	0.525
Age	−0.285	0.001
High	−0.051	0.574
Weigh	0.272	0.002
BMI	0.342	0.000
ESS	0.351	0.000
AHI	0.694	0.000
Supine AHI	0.606	0.000
LSaO_2_	−0.506	0.000
CT90	0.627	0.000

^a^ One‐way ANOVA test.

Abbreviations: AHI, apnea–hypopnea index; BMI, body mass index; CT90, the percentage of total sleep time spent with SaO_2_ < 90%; ESS, Epworth Sleepiness Scale; LSaO_2_, the lowest oxygen saturation; PAP, positive airway pressure.

Multiple linear regression analysis (Table 3) revealed that PAP pressure was associated with gender (*b* = 1.142, *p* = 0.032), age (*b* = −0.039, *p* = 0.005), AHI (*b* = 0.047, *p* = 0.000), and CT90 (*b* = 0.037, *p* = 0.000). Using these variables, we developed the following prediction formula for PAP pressure:

**TABLE 3:** Stepwise multiple regression analysis of all patients using PAP pressure as dependent variables.

Variable	*B*	*β*	95%CI	*p*
Sex	1.142	0.139	0.102, 2.182	0.032
Age, years	−0.039	−0.152	−0.066, −0.012	0.005
Weigh	NA	0.009	NA	0.900
High	NA	−0.019	NA	0.784
BMI	NA	0.036	NA	0.789
ESS	NA	0.118	NA	0.074
AHI	0.047	0.473	0.028, 0.065	0.000
Supine AHI	NA	−0.013	NA	0.927
LSaO_2_	NA	−0.056	NA	0.554
CT90	0.037	0.260	0.013, 0.060	0.003

Abbreviations: AHI, apnea–hypopnea index; BMI, body mass index; CT90, the percentage of total sleep time spent with SaO_2_ < 90%; ESS, Epworth Sleepiness Scale; LSaO_2_, the lowest oxygen saturation; PAP, positive airway pressure.

PAPpre (cmH_2_O) = 8.548 + 1.142 × sex − 0.039 × age + 0.047 × AHI + 0.037 × CT90 (*R* ^2^ = 0.553), with male coded as 0 and female as 1.

**FIGURE 3:**
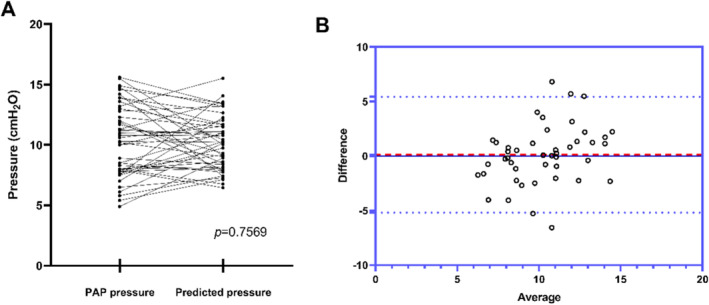
The pressure prediction formula was employed to forecast the pressure in the validation group. (A) The actual measured pressure and predicted pressure were plotted, resulting in a *p*‐value of 0.7569. (B) A different‐average diagram of actual pressure and predicted pressure was generated with a bias of 0.1258 and a 95% confidence interval ranging from −5.185 to 5.437.

To validate the prediction formula, we applied it to the 45 patients in the validation group. Figure 3 demonstrates that the predicted pressure did not significantly differ from the actual pressure (*p* = 0.7569), with a difference of 0.126 ± 2.710 cmH_2_O between them.

**FIGURE 4:**
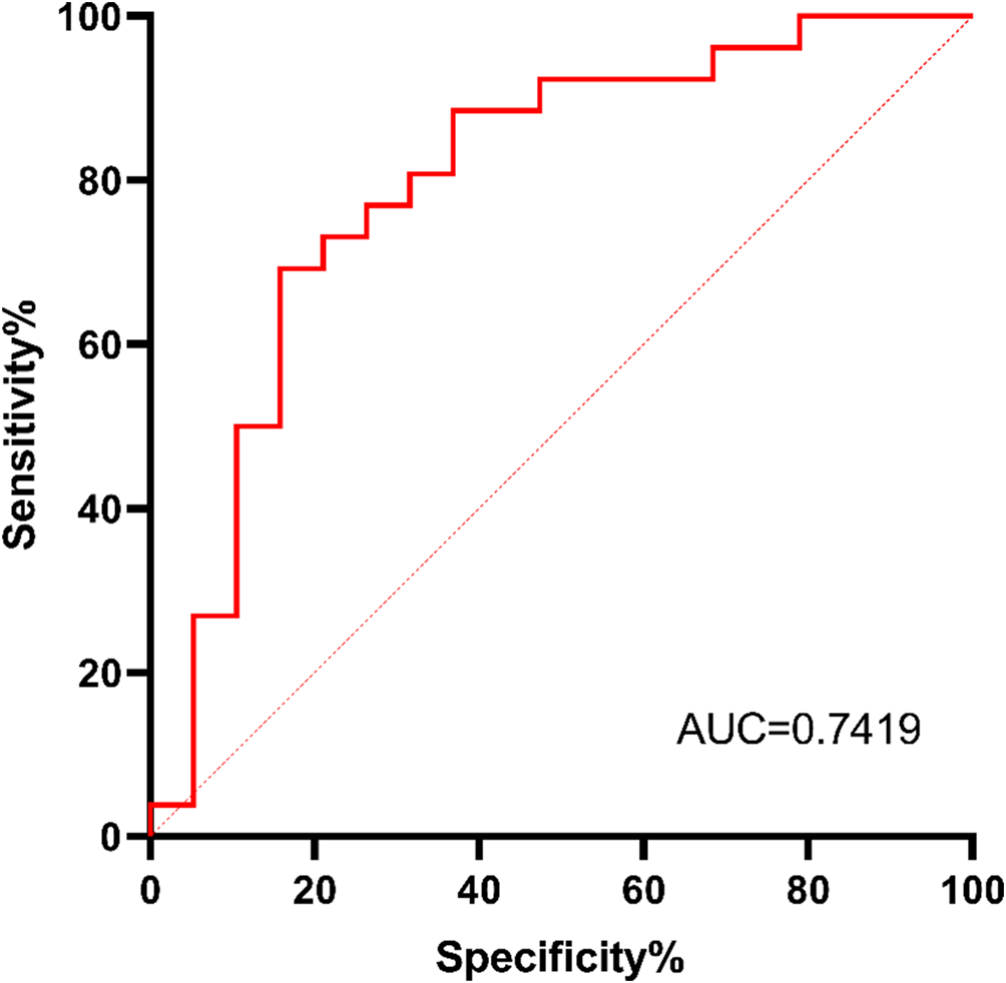
The ROC curve utilized to evaluate the discrimination performance of the PAP pressure prediction formula, with an area under the curve (AUC) of 0.7419 for PAP pressure ≥ 12 cmH_2_O.

We defined high pressure as PAP pressure ≥ 12 cmH_2_O and low pressure as PAP pressure < 12 cmH_2_O. In the validation group, we used the PAP pressure prediction formula to predict high CPAP pressure (≥ 12 cmH_2_O) sensitivity and specificity and plotted the receiver operating characteristic (ROC) curve. The area under the ROC curve (AUROC) was 0.7419, indicating good diagnostic accuracy (Figure 4).

## 4 Discussion

Our study reveals that PAP pressure is correlated with age, sex, and the severity of OSA (AHI, CT90), rather than obesity (BMI) in China. The newly developed formula exhibits high accuracy in predicting pressure, as evidenced by the ACU of 0.7419 in external data validation. Furthermore, the bias of the different‐average diagram of actual pressure and predicted pressure is 0.1258, surpassing previous studies [12]. The PAP pressure prediction formula can serve as a preliminary guide for setting APAP initial pressure or conducting CPAP titration. Additionally, for patients with high predicted pressure, pre‐emptive patient education can enhance PAP compliance [15].

Previous studies have identified the severity of OSA (AHI, ODI, or LSaO_2_) and obesity (BMI or neck circumference) as significant predictors of PAP pressure ([12], [16, 17, 18, 19]). In our study, we included CT90, an indicator of OSA severity, as a predictor of PAP pressure, consistent with prior research. CT90 reflects the duration of upper airway collapse and can indicate the severity of OSA. A higher CT90 value corresponds to a greater need for PAP pressure to open the upper airway [1]. Interestingly, our findings showed that BMI is not a significant predictor, which diverges from earlier studies [19]. This may be because CT90, the variable we used, is itself influenced by obesity, which is a critical factor that impacts OSA severity. Our univariate analysis suggested that BMI and weight were associated with predicting pressure, but in multivariate analysis, these variables were not significant, which supports our hypothesis.

Our study revealed that gender is a predictor, aligning with prior research [20, 21]. In addition, we observed a decrease in PAP pressure with increasing age, which contrasts with earlier studies [17, 18, 19]. This may be attributed to the fact that as people age, the muscles around the airway tend to relax and tension decreases, resulting in lower PAP pressure requirements [22].

However, our study has some limitations. First, the sample size was small, and there was no contingency verification. Second, because of the retrospective nature of the analysis, certain variables were not collected. Third, we utilized automatic pressure titration rather than CPAP titration, which may introduce errors in pressure measurement. Lastly, the prediction model demonstrated efficacy in group‐based predictions; however, its performance for certain individuals was less optimal, suggesting a need for additional data in future research endeavors.

## 5 Conclusion

The optimal PAP pressure in Chinese individuals can only be predicted by PSG variables and demographic characteristics (*R* ^2^ = 0.553).

## Author Contributions

All authors conceived the study design, undertook the methodology, collected data, analyzed data, and edited the manuscript. All authors reviewed and approved the final manuscript to be published.

## Ethics Statement

All procedures performed in studies involving human participants were in accordance with the ethical standards of the institutional and/or national research committee (the Human Research Ethics Committee of the Sixth Affiliated Hospital of Sun Yat‐sen University) and with the 1964 Helsinki Declaration and its later amendments or comparable ethical standards.

## Consent

As the study was retrospective in nature, individual consent from study participants was not obtained.

## Conflicts of Interest

The authors declare no conflicts of interest.

## Data Availability

The datasets generated during and/or analyzed during the current study are available from the corresponding author on reasonable request.
